# A nomogram for predicting 10-year cancer specific survival in patients with pathological T3N0M0 rectal cancer

**DOI:** 10.3389/fmed.2022.977652

**Published:** 2022-08-22

**Authors:** Shuang Liu, Shanfei Yang, Haina Yu, Huilong Luo, Gong Chen, Yuanhong Gao, Rui Sun, Weiwei Xiao

**Affiliations:** ^1^Department of Radiation Oncology, Sun Yat-sen University Cancer Center, Guangzhou, China; ^2^State Key Laboratory of Oncology in South China, Sun Yat-sen University Cancer Center, Guangzhou, China; ^3^Collaborative Innovation Center for Cancer Medicine, Sun Yat-sen University Cancer Center, Guangzhou, China; ^4^Department of Colorectal Surgery, Sun Yat-sen University Cancer Center, Guangzhou, China

**Keywords:** pT3N0M0, rectal cancer, nomogram, CSS, adjuvant chemotherapy

## Abstract

**Background:**

The pathological T3N0M0 (pT3N0M0) rectal cancer is the earliest stage and has the best prognosis in the locally advanced rectal cancer, but the optimal treatment remains controversial. A reliable prognostic model is needed to discriminate the high-risk patients from the low-risk patients, and optimize adjuvant chemotherapy (ACT) treatment decisions by predicting the likelihood of ACT benefit for the target population.

**Patients and methods:**

We gathered and analyzed 276 patients in Sun Yat-sen University Cancer Center from March 2005 to December 2011. All patients underwent total mesorectal excision (TME), without preoperative therapy, and were pathologically proven pT3N0M0 rectal cancer with negative circumferential resection margin (CRM). LASSO regression model was used for variable selection and risk factor prediction. Multivariable cox regression was used to develop the predicting model. Optimum cut-off values were determined using X-Tile plot analysis. The 10-fold cross-validation was adopted to validate the model. The performance of the nomogram was evaluated with its calibration, discrimination and clinical usefulness.

**Results:**

A total of 188 patients (68.1%) had ACT and no patients had adjuvant radiotherapy. Age, monocyte percentage, carbohydrate antigen 19–9, lymph node dissection numbers and perineural invasion (PNI) were identified as significantly associated variables that could be combined for an accurate prediction risk of Cancer Specific Survival (CSS) for pT3N0M0 patients. The model adjusted for CSS showed good discrimination with a C-index of 0.723 (95% CI: 0.652–0.794). The calibration curves showed that the nomogram adjusted for CSS was able to predict 3-, 5-, and 10-year CSS accurately. The corresponding predicted probability was used to stratify high and low-risk patients (10-year CSS: 69.1% vs. 90.8%, HR = 3.815, 95%CI: 2.102–6.924, *P* < 0.0001). ACT improved overall survival (OS) in the low-risk patients (10-year OS: 91.9% vs. 83.3%, HR = 0.338, 95% CI: 0.135–0.848, *P* < 0.0001), while it did not exhibit a significant benefit in the high-risk patients.

**Conclusion:**

The present study showed that age, monocyte percentage, carbohydrate antigen 19–9, lymph node dissection numbers and PNI were independent prognostic factors for pT3N0M0 rectal cancer patients. A nomogram based on these prognostic factors effectively predicts CSS in patients, which can be conveniently used in clinical practice. ACT may improve overall survival in the low-risk patients. But the benefit of ACT was not seen in the high-risk patients.

## Background

The pathological T3N0M0 (pT3N0M0) rectal cancer is the earliest stage and the best prognosis in the locally advanced rectal cancer. Although neoadjuvant chemoradiotherapy plus interval chemotherapy is the treatment for clinical T3N0M0 patients recommended by the guidelines (1), many patients do not receive neoadjuvant therapy due to the adverse symptoms and high costs. In addition, due to the inaccuracy of the image, some patients who were diagnosed clinical T1-2 might be confirmed T3 after total mesorectal excision (TME) surgery. For pT3N0M0 patients, positive circumferential resection margin (CRM) after surgery was considered to be an independent prognostic factor of clinical outcome (2, 3), and these patients require adjuvant chemoradiotherapy. As for pT3N0M0 patients with negative CRM, there has been little data to guide recommendations in adjuvant chemoradiotherapy. It was crucial to optimize and guide personalized treatment through further effective risk stratification for these patients.

Although it is difficult to stratify patients based on the traditional TNM staging system for pT3N0M0 rectal cancer with negative resection margin, clinical features and several potential prognostic factors warrant further studies. For the TNM staging system, the N factor depends on whether there is regional lymph node metastasis and the extent of metastasis The inadequate lymph nodes dissection examined results in an inaccurate number of positive lymph nodes and inaccurate staging (4, 5). Current guidelines especially recommended that at least 12 lymph nodes be resected and histologically evaluated (6–8). Furthermore, the adjuvant chemotherapy (ACT) regimen is determined by the number of lymph node dissection metastases (9).

The prognosis of rectal cancers is correlated with host- and tumor-related factors (10–12). Peripheral blood monocyte was one of the well-known indicators of the immune status of cancer patients (13, 14). Existing studies suggest that a decreased lymphocyte-to-monocyte ratio before treatment was independently associated with worse overall survival in rectal cancers who underwent surgery (15). Pretreatment lymphocyte count was also independently associated with ACT efficacy for the high-risk patients in Stage II rectal cancers (16). Further, we attempted to investigate other potential blood biomarkers and pathologic conditions included surgical margin status, tumor thrombus, perineural invasion (PNI) of patients’ related disease outcomes in this study.

A previous study based on the Surveillance, Epidemiology, and End Results (SEER) database developed a risk-stratification model for pT3N0 rectal patients, which was composed of age, tumor differentiation, and the number of nodes resected (17). The long-term survival was better for low-risk patients than for high-risk patients (5-year CSS: 92.13% vs. 72.55%, *P* < 0.001). The sequential radiotherapy after surgery doubled 5-year CSS in high-risk patients (42.06% vs. 91.26%, *P* = 0.001), while showed no obvious survival benefit in the low-risk patients (93.36% vs. 96.38%, *P* = 0.182). The model may not be able to inaccurately predict clinical outcomes in the Chinese population because of ethnical diversity and environmental exposures. The 5-year CSS of overall cohort from SEER database was 86.31%, while the 5-year CSS in our study was 89.5%.

In this study, we combined the common clinical variables with potential prognostic indicators to develop a nomogram to predict CSS in patients with pT3N0M0 rectal cancer with negative CRM. We aimed to provide clinicians with more guidance to stratify those high-risk score patients who need more comprehensive treatment and closer follow-up to improve survival.

## Patients and methods

### Patients

We collected retrospective data about 276 patients with rectal cancer who underwent standard TME at Sun Yat-sen University Cancer Center from March 2005 to December 2011. The inclusion criteria were as follows: (1) pathological diagnosis of rectal cancer; (2) postoperative pathological stage of pT3N0M0; (3) complete surgical resection; and (4) no preoperative therapy. Patients were excluded if they died of postoperative complications within 30 days or with positive surgical margins. This research was approved by the Ethical Committee of Sun Yat-sen University Cancer Center (B2022-005-01), and written informed consent was obtained from participants for the use of their clinical records in this study.

### Preoperative examination and assessment

All eligible patients received complete preoperative evaluation. The clinical TNM (8th edition) stage was assessed according to endorectal ultrasound (EUS) and magnetic resonance imaging (MRI) and computed tomography (CT) scan in all patients.

The laboratory tests including routine blood tests, blood biochemistry, and assessment for tumor markers [carcinoembryonic antigen (CEA) and carbohydrate antigen 19-9 (CA199) level]. To exclude the influence of various comorbidities or other disease states, all included patients had no self-reported acute infections or colorectal disorders, indicating that the hematologic markers could represent the baseline value.

### Surgical specimen and pathological assessment

Surgical resection was defined as radical when there was no evidence of distant metastases and tumor clearance was both macroscopically and histologically complete. All operations are performed by experienced colorectal surgeons in accordance with TME principles, and the surgical approaches included Hartmann, Dixon, and Miles surgery.

Two pathologists who were blinded to the clinical outcomes of the patients assessed all the resection specimens according to the eighth edition of the American Joint Committee on Cancer (AJCC) TNM staging category. Pathologic assessment included surgical margin status, tumor thrombus, PNI, positive lymph node numbers, and lymph node dissection numbers (LNDs).

### Follow-up

The first follow-up evaluation was underwent 3–5 weeks after TME surgery. Follow-up after surgery was conducted every 3–6 months for the first 2 years after treatment, every 6 months for next 3 years, and annually after 5 years. Follow-up data were obtained from medical records, telephone calls, and the population death information registration system. CSS was defined as the time from initial diagnosis until the date of cancer-specific death. OS was calculated from initial diagnosis to death due to any cause or the last follow-up.

### Statistical analysis

The least absolute shrinkage and selection operator (LASSO) method for features selection in Cox regression analysis was used to determine the most meaningful predictive clinicopathological factors. Statistical significance was defined as *P* < 0.05. X-tile software (Version 3.6.1) was used to determine the optimal cut-off values for continuous variables, including age, CA199, monocyte percentage (MONO%), and LNDs (18). The optimal cut-off values were 67 years for age, 27 U/ml for CA199, 7.6% for MONO%, and 12 for LNDs according to the X-tile software recommendation.

Nomogram model was utilized to generate the probability of 3, 5, and 10-year CSS. The ‘‘rms’’ package^1^ within R project was utilized for nomogram model building and visualization. Candidate models were constructed for all possible feature combinations, and the final model with the highest C-index was chosen. Internal validation of the model was evaluated by bootstrapping using 1,000 samples. Calibration curves for 3-, 5-, and 10-year CSS were drawn to investigate the closeness between predicted survival and the actual survival. According to the nomogram model, we calculated the total points of each patient were by plus point from each characteristic. We further classified the patients into high-risk subgroup and low-risk subgroup based on the total points. All statistical tests in this study were performed in IBM SPSS statistics (Version 23.0), R project (Version 3.6.0), and X-tile (Version 3.6.1).

## Results

### Clinicopathologic characteristics of patients

A total of 276 patients were included in this study, 68.1% (*n* = 188) received ACT and 31.9% (*n* = 88) had no adjuvant chemotherapy (non-ACT). 138 patients received single-agent ACT (5-FU/LV: 5-fluorouracil/leucovorin or capecitabine) and 50 patients received multi-agent ACT (FOLFOX: 5-fluorouracil with oxaliplatin or Capeox: capecitabine with oxaliplatin). Fifty-one patients received ACT for 3 months or less, and 137 patients received ACT for more than 3 months. The mean duration of follow-up was 141.02 ± 6.12 years. The 10-year OS was 78.6% (217/276) and the 10-year CSS was 81.2% (224/276) for the whole population. Table 1 presents the clinicopathologic characteristics of all patients. Of all patients, 61.6% (*n* = 170) of patients were female and 67.8% (*n* = 187) patients were aged ≤ 67 years. The patients with low rectal cancer (distance to anal verge less than 5 cm) were 23.9% (*n* = 66). 79.3% (*n* = 219) patients received Dixon surgery, and 18.1% (*n* = 50) patients received Miles surgery.

**TABLE 1:** Baseline clinicopathologic characteristics of the patients with pT3N0M0 rectal cancer.

Characteristics	Total, n (%)	non-ACT, n (%)	ACT, n (%)	*P-value*
Total	276 (100%)	88 (31.9%)	188 (68.1%)	
Gender				1
Female	170 (61.6%)	54 (61.4%)	116 (61.7%)	
Male	106 (38.4%)	34 (38.6%)	72 (38.3%)	
Age				0.005
≤67 years	187 (67.8%)	49 (55.7%)	138 (73.4%)	
>67 years	89 (32.2%)	39 (44.3%)	50 (26.6%)	
Distance to anal verge				0.457
≤5 cm	66 (23.9%)	24 (27.3%)	42 (22.3%)	
>5 cm	210 (76.1%)	64 (72.7%)	146 (77.7%)	
Monocyte percentage (MONO%)				0.61
≤7.6%	152 (55.1%)	46 (52.3%)	106 (56.4%)	
>7.6%	124 (44.9%)	42 (47.7%)	82 (43.6%)	
Carcinoembryonic antigen (CEA)				0.034
Normal (≤ 5 ng/ml)	171 (62.0%)	63 (71.6%)	108 (57.4%)	
Elevated (> 5 ng/ml)	105 (38.0%)	25 (28.4%)	80 (42.6%)	
Carbohydrate antigen 19-9 (CA199)				0.01
≤27 U/ml	225 (81.5%)	80 (90.9%)	145 (77.1%)	
>27 U/ml	51 (18.5%)	8 (9.09%)	43 (22.9%)	
Surgery approach				0.958
Dixon	219 (79.3%)	71 (80.7%)	148 (78.7%)	
Miles	50 (18.1%)	15 (17.0%)	35 (18.6%)	
Hartmann	7 (2.54%)	2 (2.27%)	5 (2.66%)	
Lymph node dissection numbers (LNDs)				0.917
≤12	122 (44.2%)	38 (43.2%)	84 (44.7%)	
>12	154 (55.8%)	50 (56.8%)	104 (55.3%)	
Perineural invasion (PNI)				0.257
Negative	183 (66.3%)	63 (71.6%)	120 (63.8%)	
Positive	93 (33.7%)	25 (28.4%)	68 (36.2%)	

ACT was more common among patients aged ≤ 67 years than among patients aged > 67 years (*P* = 0.005), among patients with CEA > 5 ng/ml than among those with ≤ 5 ng/ml (*P* = 0.034), and among patients with CA199 > 27 U/ml than among those with ≤ 27 U/ml (*P* = 0.01).

### Independent prognostic factors of cancer specific survival

Univariate analysis was performed on all collected variables. The results revealed that age (*P* = 0.005), monocyte percentage (MONO%) (*P* < 0.001), carbohydrate antigen 19-9 (CA199) (*P* = 0.003), lymph node dissection numbers (LNDs) (*P* = 0.004), and PNI (*P* = 0.004) were considered significant predictors for CSS (Table 2). Multivariate Cox regression analysis showed age (HR = 1.877, 95%CI: 1.085–3.249, *P* = 0.024), MONO% (HR = 2.496, 95%CI: 1.415–4.403, *P* = 0.002), CA199 (HR = 2.306, 95%CI: 1.284–4.142, *P* = 0.005), LNDs (HR = 0.442, 95%CI: 0.251–0.778, *P* = 0.005), and PNI (HR = 2.126, 95%CI: 1.244–3.632, *P* = 0.006) were significantly associated with CSS (Table 2).

**TABLE 2:** Prognostic factors of 10-year CSS in univariate analysis and multivariate analysis.

Characteristics	10-year CSS	Univariate analysis		Multivariate analysis	
		HR (95% CI)	*P-value*	HR (95% CI)	*P-value*
Gender (Male vs. Female)		0.608 (0.339–1.090)	0.095		
Age (≤67 years vs. > 67 years)	85.6% vs. 71.9%	2.140 (1.254–3.651)	0.005	1.877 (1.085–3.249)	0.024
Distance to anal (≤5 cm vs. > 5 cm)	84.4% vs. 80.0%	1.118 (0.589–2.124)	0.733		
Monocyte percentage (MONO%) (≤7.6% vs. > 7.6%)	88.2% vs. 72.6%	2.510 (1.435–4.390)	0.001	2.496 (1.415–4.403)	0.002
Carcinoembryonic antigen (CEA) (≤5 ng/ml vs. > 5 ng/ml)	82.5% vs. 79.9%	1.340 (0.784–2.293)	0.285		
Carbohydrate antigen 19-9 (CA199) (≤ 27 U/ml vs. > 27 U/ml)	84.4% vs. 66.7%	2.417 (1.359–4.296)	0.003	2.306 (1.284–4.142)	0.005
Surgery approach					
Dixon	81.3%	—			
Miles	82.0%	1.150 (0.591–2.239)	0.680		
Hartmann	71.4%	1.998 (0.482–8.275)	0.340		
Lymph node dissection numbers (LNDs) (≤12 vs. > 12)	74.6% vs. 86.4%	0.444 (0.257–0.767)	0.004	0.442 (0.251–0.778)	0.005
Perineural invasion (PNI) (Negative vs. Positive)	86.3% vs. 71%	2.182 (1.279–3.722)	0.004	2.126 (1.244–3.632)	0.006
Adjuvant chemotherapy (ACT)	79.5% vs. 81.9%	0.809 (0.463–1.416)	0.459		

### Subgroup analysis

**FIGURE 1:**
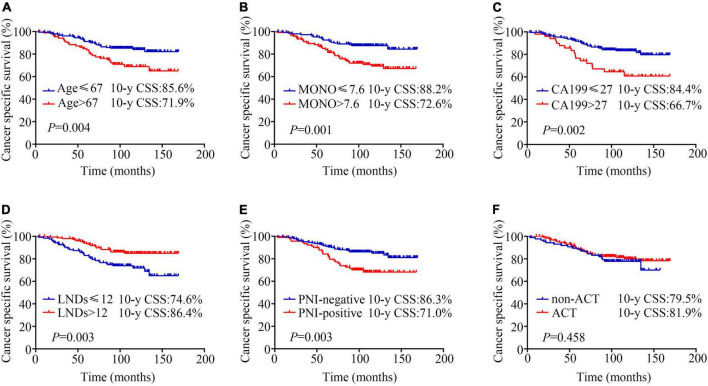
Kaplan-Meier analysis of cancer specific survival according to **(A)** age (≤67 years vs. > 67 years, 10-y CSS: 85.6% vs. 71.9%, *P* = 0.004); **(B)** monocyte percentage (MONO%) (≤ 7.6% vs. > 7.6%, 10-year CSS: 88.2% vs. 72.6%, *P* = 0.001); **(C)** carbohydrate antigen 19-9 (CA199) (≤27 U/ml vs. > 27 U/ml, 10-year CSS: 84.4% vs. 66.7%, *P* = 0.002); **(D)** lymph node dissection numbers (LNDs) (≤ 12 vs. > 12, 10-year CSS: 74.6% vs. 86.4%, *P* = 0.003); **(E)** perineural invasion (PNI) (negative vs. positive, 10-year CSS: 86.3% vs. 71%, *P* = 0.003); **(F)** adjuvant chemotherapy (ACT) (non-ACT vs. ACT, 10-year CSS: 79.5% vs. 81.9%, *P* = 0.458).

Kaplan-Meier analysis showed that patients aged ≤ 67 years had a better prognosis than those aged > 67 years (10-year CSS: 85.6% vs. 71.9%, *P* = 0.004; Figure 1A). The patients with MONO% ≤ 7.6% had better outcomes than patients with MONO% > 7.6% (10-year CSS: 88.2% vs. 72.6%, *P* = 0.001; Figure 1B). The patients with CA199 ≤ 27 U/ml had an obvious advantage in survival than patients with CA199 > 27 U/ml (10-year CSS: 84.4% vs. 66.7%, *P* = 0.002; Figure 1C). The patients for whom ≤ 12 nodes had been resected had a poorer prognosis than patients for whom > 12 nodes had been resected (10-year CSS: 74.6% vs. 86.4%, *P* = 0.003; Figure 1D). The positive PNI was detrimental for patient survival (10-year CSS: negative vs. positive, 86.3% vs. 71.0%, *P* = 0.003; Figure 1E). However, no survival difference was observed between the patients who received ACT and those who did not (10-year CSS: 81.9% vs. 79.5%, *P* = 0.523; Figure 1F). These variables were also assessed when using OS as an endpoint. Similar findings were obtained (Supplementary Figures 1A–F).

### Construction and internal validation of the nomogram for cancer specific survival

**FIGURE 2:**
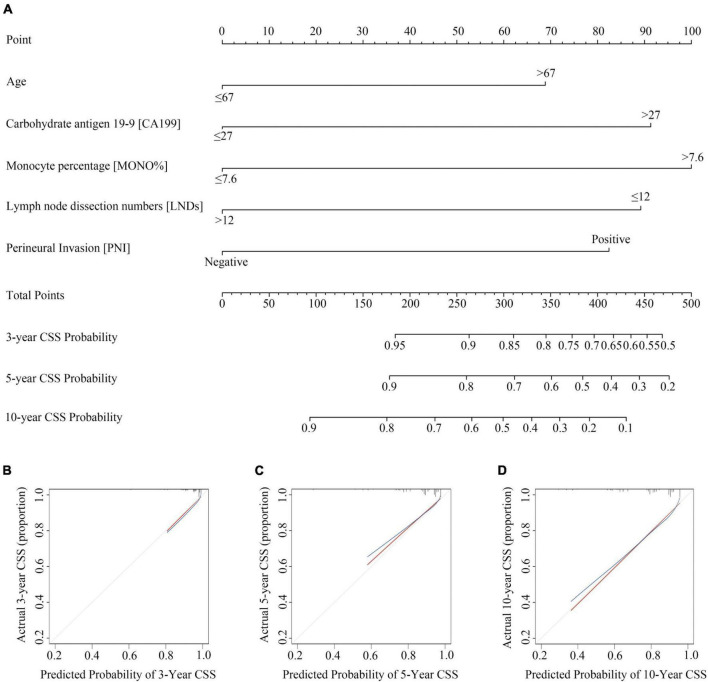
Construction and validation of Nomogram for cancer specific survival probability in pT3N0M0 rectal cancer patients. **(A)** The nomogram was developed with age, monocyte percentage (MONO%), carbohydrate antigen 19-9 (CA199), lymph node dissection numbers (LNDs), and perineural invasion (PNI); **(B–D)** calibration curves of the CSS nomogram, indicating the consistency between predicted and observed 3-, 5-, and 10-year outcomes.

Based on the results of the LASSO regression and multivariate COX regression, the nomogram incorporating five predictors was established to predict CSS in pT3N0M0 rectal cancer patients following TME surgery (Figure 2A). According to our nomogram plot, total points of each pT3N0M0 rectal cancer patients was calculated as follows: Age > 67 years (69 points), CA199 > 27 U/ml (91 points), MONO% > 7.6% (100 points), LNDs ≤ 12 (89 points), and positive PNI (82 points). Each of these variables was assigned a score based on the point scale. By adding up the total point from all the variables, we could estimate 3-, 5-, and 10-year CSS probability. The C-index for the nomogram model was 0.723 (95% CI: 0.652–0.794). The calibration plots for 3-, 5-, and 10-year CSS probability also exhibited good internal consistency between the predicted CSS and the actual CSS (Figures 2B–D).

**FIGURE 3:**
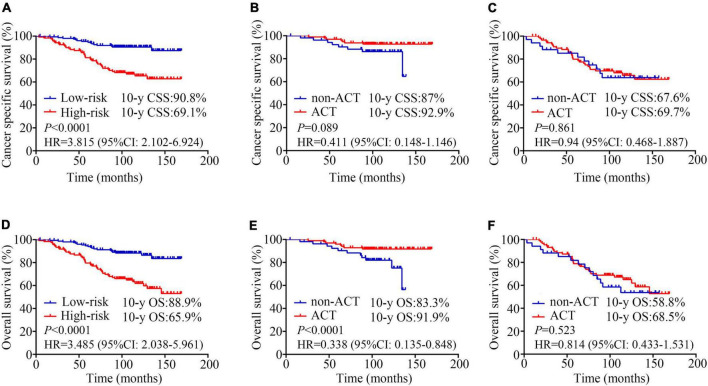
Kaplan-Meier analysis estimates. Cancer specific survival according to **(A)** risk stratifications (low-risk vs. high-risk, 10-year CSS: 69.1% vs. 90.8%, HR = 3.815, 95%CI: 2.102–6.924, *P* < 0.0001); **(B)** adjuvant chemotherapy (ACT) for low-risk patients (non-ACT vs. ACT, 10-year CSS: 92.9% vs. 87%, HR = 0.411, 95% CI: 0.148–1.146, *P* = 0.089); **(C)** adjuvant chemotherapy (ACT) for high-risk patients (non-ACT vs. ACT, 10-year CSS: 67.6% vs. 69.7%, HR = 0.94, 95% CI: 0.468–1.887, *P* = 0.861); Overall survival according to **(D)** risk stratifications (low-risk vs. high-risk, 10-year OS: 65.9% vs. 88.9%, HR = 3.485, 95%CI: 2.038–5.961, *P* < 0.0001); **(E)** adjuvant chemotherapy (ACT) for low-risk patients (non-ACT vs. ACT, 10-year OS: 91.9% vs. 83.3%, HR = 0.338, 95% CI: 0.135–0.848, *P* < 0.0001); **(F)** adjuvant chemotherapy (ACT) for high-risk patients (non-ACT vs. ACT, 10-year OS: 58.8% vs. 68.5%, HR = 0.814, 95% CI: 0.433–1.531, *P* = 0.523).

We used total point = 170, corresponding to a 5-year CSS probability of 80%, as a cut-off value to stratify patients. In our study, 123 patients with total point > 170 were classified as high-risk group (44.6%), and 153 patients with total point ≤ 170 were classified as low-risk group (55.4%). CCS was significantly higher in the low-risk group than in the high-risk group (10-year CSS: 69.1% vs. 90.8%, HR = 3.815, 95%CI: 2.102–6.924, *P* < 0.0001; Figure 3A). OS was also significantly higher in the low-risk group than in the high-risk group (10-year OS: 65.9% vs. 88.9%, HR = 3.485, 95%CI: 2.038–5.961, *P* < 0.0001; Figure 3D). And our model was superior to the previous model (17) (10-year CSS: 87.8% vs. 74.8%, HR = 2.445, 95%CI: 1.353–4.418, *P* = 0.003; 10-year OS: 87.8% vs. 69.6%, HR = 2.971, 95% CI: 1.696–5.204, *P* < 0.0001; Supplementary Figures 2A,B).

In the low-risk patients, the application of ACT could benefit the patient’s survival (10-year CSS: 92.9% vs. 87%, HR = 0.411, 95% CI: 0.148–1.146, *P* = 0.089; 10-year OS: 91.9% vs. 83.3%, HR = 0.338, 95% CI: 0.135–0.848, *P* < 0.0001; Figures 3B,E). However, no survival difference was observed between high−risk patients who treated with surgery plus ACT vs. those who treated with surgery alone (10-year CSS: 67.6% vs. 69.7%, HR = 0.94, 95% CI: 0.468–1.887, *P* = 0.861; 10-year OS: 58.8% vs. 68.5%, HR = 0.814, 95% CI: 0.433–1.531, *P* = 0.523; Figures 3C,F).

Further analysis showed that there was no significant difference in survival between single-agent chemotherapy and multi-agent chemotherapy regimen (10-year CSS: 71.6% vs. 63.6%, HR = 1.609, 95%CI: 0.641–4.039, *P* = 0.311; 10-year OS: 70.1% vs. 63.6%, HR = 1.438, 95% CI: 0.586–3.529, *P* = 0.380); Supplementary Figures 3A,C). There was no apparent survival benefit between 3 months or less of ACT vs. more than 3 months of ACT (10-year CSS: 72.2% vs. 69.0%, HR = 1.095, 95%CI: 0.426–2.819, *P* = 0.850; 10-year OS: 72.2% vs. 67.6%, HR = 1.145, 95% CI: 0.453–2.894, *P* = 0.783; Supplementary Figures 3B,D).

## Discussion

Firstly, to our knowledge, this was the first and only study of a nomogram for predicting CSS in patients with pT3N0M0 rectal cancer. Secondly, this model is established by screening the complete common preoperative laboratory test indicators and pathological outcomes, which makes it more accurate and more targeted. Age, MONO%, CA199, LNDs, and PNI were performed as independent factors to construct our prognostic nomogram model. Our nomogram had good discrimination (C-index = 0.723, 95% CI: 0.652–0.794; Figure 2A), which provided a convenient and feasible tool for predicting the risk of pT3N0M0 patients. Thirdly, our model effectively distinguishes high-risk and low-risk groups, which could guide the choice of postoperative treatment for T3N0M0 colorectal cancer patients. For Chinese patients, our nomogram (10-year CSS: 69.1% vs. 90.8%, HR = 3.815, 95%CI: 2.102–6.924, *P* < 0.0001; 10-year OS: 65.9% vs. 88.9%, HR = 3.485, 95%CI: 2.038–5.961, *P* < 0.0001; Figures 3A,D) had a better prediction effect than the previous risk-stratification model (17) (10-year CSS: 87.8% vs. 74.8%, HR = 2.445, 95%CI: 1.353–4.418, *P* = 0.003; 10-year OS: 87.8% vs. 69.6%, HR = 2.971, 95% CI: 1.696–5.204, *P* < 0.0001; Supplementary Figures 2A,B).

Over the years, several studies have focused on the role of adjuvant treatment including chemotherapy and radiotherapy in T3N0 patients (17, 19–21). Luke C. Peng et al. collected 4,724 patients with T3N0M0 rectal cancer diagnosed between 1998 and 2008 in the SEER database. The results demonstrated that adjuvant radiotherapy was significantly associated with improved CSS compared with surgery alone (HR = 0.688, 95% CI: 0.578–0.819, *P* < 0.001), while neoadjuvant radiotherapy had no significant benefits (HR = 0.863, 95% CI: 0.715–1.043, *P* = 0.127) (19). Another study reported that postoperative concurrent chemoradiotherapy could significantly decrease locoregional recurrence rate in patients with CRM- but having one risk factor (distance from anal verge ≤ 5 cm or distal resection margin ≤ 2 cm) (5-year locoregional recurrence free survival: 98.9% vs. 87.4%, *P* = 0.006) (21). Unlike these prior studies, our study focused on the effect of ACT alone. In the patients who were classified as low-risk patients according to this model (total points ≤ 170), the application of ACT after surgery could benefit the patient’s survival (10-year OS: 91.9% vs. 83.3%, HR = 0.338, 95% CI: 0.135–0.848, *P* < 0.0001; Figure 3E), whereas high-risk patients had no significant survival benefit (10-year OS: 58.8% vs. 68.5%, HR = 0.814, 95% CI: 0.433–1.531, *P* = 0.523; Figure 3F). ACT alone might be insufficient for high-risk patients, and the combination of radiotherapy and chemotherapy should be considered.

In high-risk stage II colon cancer, the addition of oxaliplatin to fluoropyrimidine did not improve overall survival (22). For duration of ACT, the IDEA study showed that 6 months of ACT was not superior to 3 months of ACT in high-risk stage II colon cancer patients [5-year DFS: 3 months group vs. 6 months group = 80.7% vs. 83.9%, HR = 1.17, 80%CI: 1.05–1.31, P (for non-inferiority) = 0.39] (23, 24) Similar to these results, we did not find a population of high-risk patients who benefited from adjuvant therapy and regimens after grouping according to the existing model. Therefore, risk stratification by identifying precise predictors of adjuvant therapy benefit in the context of patient individualization is necessary for high-risk patients. Mismatch repair (MMR) status may be considered one of the most powerful prognostic indicators (25). Detection of circulating tumor DNA (ctDNA) is also currently considered a useful tool to guide the application of ACT (26).

The TNM system is based on the depth of invasion, distant metastases and the number of positive lymph nodes. Indeed, accumulating studies have noted the predictive value of the lymph node status and lymph node ratio (LNR) in CRC. Notably, the 5-year survival of patients with negative lymph nodes (80%) was significantly higher than that (45%) of those with positive nodes (*P* < 0.05) (27). While the number of retrieved LNs is influenced by various factors including age and gender, the experience or skill of the surgeon and even the ethnicity of patients. Kidner et al. found that the 5-year survival rate of stage I/II patients with 1–4 lymph nodes removed was 48%, while that of patients with more than 20 lymph nodes removed was 65% (28). Another survival analysis determined that in patients with CRC without metastatic lymph nodes, the CSS of patients with 1–11 lymph nodes removed was significantly worse than that of patients with more than 12 lymph nodes removed (CSS: 62.3% vs. 75.1%, HR = 0.59, 95%CI: 0.41–0.84, *P* = 0.004) (29). In addition, Sarli et al. found that patients with no more than 9 lymph nodes examined have a similar 5-year survival rate to patients with 1–3 positive lymph nodes (51.3% vs. 52.6%), and postoperative chemotherapy recommend for N0 patients with only a few nodes examined (30). The NCCN and AJCC/UICC guidelines recommend at least 12 lymph nodes should be examined as the current standard for pathological examination in CRC surgery (31). Reviewing the presented data, 12 assessable lymph nodes retrieved as adequate lymph node count could be a biomarker to evaluate the prognosis of pT3N0M0 rectal cancer. In our study, the 12 lymph nodes minimum for adequacy was achieved in over 50% of the total study cohort, and in the previous study, the rate ranged from 36 to 67% (32).

The expression of CA 199 occurs as a result of the presence of sialylated Lewis a blood group antigen, is a tumor-associated antigen elevated in many types of cancer (33). CA199 levels have also been demonstrated to be predictive of malignancy in numerous previous studies (34, 35). Several studies have expressed concern that it was one of the best available prognostic indicators in colorectal cancers (36, 37). Especially, Zheng et al. showed that a higher level of serum levels of CA19-9 may serve as a useful marker effective in identifying node-negative CRCs had a poor prognosis after surgery and chemotherapy (38).

PNI can occur when neoplastic cells are missed since they can travel along nerves far from the primary lesion. This hinders surgery’s ability to establish local control over malignancy (39, 40). PNI is a strong prognostic factor for colorectal cancer, which is generally associated with worse oncological outcomes. In the eighth edition of TNM, PNI was introduced as a supporting factor (41). This study showed that PNI was an independent prognostic factor for cancer-specific survival in multivariate analysis (HR = 2.126, 95% CI: 1.244-3.632, *P* = 0.006) (42). A comprehensive meta-analysis has also shown that PNI was an independent prognostic factor for 5-year overall, 5-year disease-free, and 5-year cancer-specific survival in multivariate analysis (HR = 1.85, 95% CI: 1.63–2.12; HR = 2.35, 95% CI: 1.97–3.08; and HR = 1.91, 95% CI: 1.50–2.42, respectively) (42). And in our study, the overall incidence of PNI was found to be 33.7%, similar to the 33% found in a previous review (43).

Recent research revealed that cancer-associated inflammation may play an important play in rectal cancer progression and prognosis (44–46). Guo et al. proposed that inflammatory cells included monocyte and related cytokines infiltrate in the tumor microenvironment, which promotes tumor angiogenesis and proliferation, survival, and migration (47). In Hu et al. study, higher peripheral monocyte counts as a useful predictor of postoperative prognosis in CRC patients and were associated with a worse 5-year disease-free survival rate (48). An elevated preoperative peripheral blood monocyte count might reflect a high degree of immune suppression and high levels of inflammatory cytokines. Similar to our results, Liu et al. constructed and validated a nomogram included monocyte count (cut off value 0.43 in the validation set) to predict individual survival probability for stage II–III colorectal cancer (49).

This study is subject to two major limitations. First, it was a retrospective study involving a single institution. Second, it would be not feasible to perform an external validation cohort study limited by sample size now. Further study should focus on validating the model by building multicenter normalized database, which includes complete laboratory examination and pathological outcomes. It should be noted that the reference standards of the variables may be slightly different in multicenter data, due to the application of different equipment and technologies in different centers. The model may have to be carefully optimized by slightly adjusting the cut-off values of some variables.

## Conclusion

Regardless of the above limitations, our study showed that age, preoperative monocyte percentage, preoperative CA199, lymph node dissection numbers, and PNI were considered significant predictors for CSS. ACT was associated with improved survival compared with TME alone in the low-risk patients. Appropriate intensive treatment and follow-up may improve the efficacy of treatment and survival for high-risk patients.

## Data availability statement

The raw data supporting the conclusions of this article will be made available by the authors, without undue reservation.

## Ethics statement

This research was approved by the Ethical Committee of Sun Yat-sen University Cancer Center (B2022-005-01). The patients/participants provided their written informed consent to participate in this study.

## Author contributions

WX, RS, and SL helped to design and refine study strategy. SL and SY screened selected studies for inclusion and extracted relevant data. SL and HY carried out data collection and analysis. SL, SY, and HY contributed to the first draft of the manuscript. WX, RS, GC, and YG supervised the project and provided critical review of the manuscript. All authors were involved in the interpretation of data and results, contributed to the revision and refinement of the final manuscript, had full access to all study data, given final approval of this version of the manuscript to be published, and agreed to be accountable to all aspects of the work, and read and approved the final manuscript.

## Footnotes

1
https://cran.r-project.org/web/packages/rms/index.html

## Supplementary material

Supplementary Figure 1 Kaplan-Meier analysis of overall survival according to **(A)** age (≤ 67 vs. > 67 years, 10-y OS: 85.6% vs. 64%, *P* < 0.0001); **(B)** monocyte percentage (MONO%) (≤ 7.6% vs. > 7.6%, 10-year OS: 96.8% vs. 68.5%, *P* < 0.0001); **(C)** carbohydrate antigen 19-9 (CA199) (≤27 U/ml vs. > 27 U/ml, 10-year OS: 81.8% vs. 64.7%, *P* = 0.003); **(D)** lymph node dissection numbers (LNDs) (≤12 vs. > 12, 10-year OS: 72.1% vs. 83.8%, *P* = 0.005); **(E)** perineural invasion (PNI) (negative vs. positive, 10-year OS: 83.1% vs. 69.9%, *P* = 0.013); **(F)** adjuvant chemotherapy (ACT) (non-ACT vs. ACT, 10-year OS: 73.9% vs. 80.9%, *P* = 0.113).

Supplementary Figure 2 Kaplan-Meier analysis estimates of the previous risk-stratification model (17) in our patients. **(A)** Cancer specific survival according to risk stratifications (low-risk vs. high-risk, 10-year CSS: 87.8% vs. 74.8%, HR = 2.445, 95%CI: 1.353–4.418, *P* = 0.003); **(B)** overall survival according to risk stratifications (low-risk vs. high-risk, 10-y OS: 87.8% vs. 69.6%, HR = 2.971, 95% CI: 1.696–5.204, *P* < 0.0001).

Supplementary Figure 3 Kaplan-Meier analysis estimates for high-risk patients. Cancer specific survival according to **(A)** adjuvant chemotherapy (ACT) for single-agent chemotherapy vs. multi-agent chemotherapy regimen, (10-year CSS: 71.6% vs. 63.6%, HR = 1.609, 95%CI: 0.641–4.039, *P* = 0.311); **(B)** ACT for 3 months or less (≤3 months) vs. more than 3 months (>3 months), (10-year CSS: 72.2% vs. 69.0%, HR = 1.095, 95%CI: 0.426–2.819, *P* = 0.850); Overall survival according to **(C)** ACT for single-agent chemotherapy vs. multi-agent chemotherapy regimen, 10-year OS: 70.1% vs. 63.6%, HR = 1.438, 95% CI: 0.586–3.529, *P* = 0.380); **(D)** ACT for ≤ 3 months vs. >3 months, (10-year OS: 72.2% vs. 67.6%, HR = 1.145, 95% CI: 0.453–2.894, *P* = 0.783).
