# Supplementary figures and images for: A nomogram for predicting 10-year cancer specific survival in patients with pathological T3N0M0 rectal cancer
Source: Front Med (Lausanne). 2022 Aug 22;9:977652. doi: 10.3389/fmed.2022.977652 (PMC9441689; doi:10.3389/fmed.2022.977652)

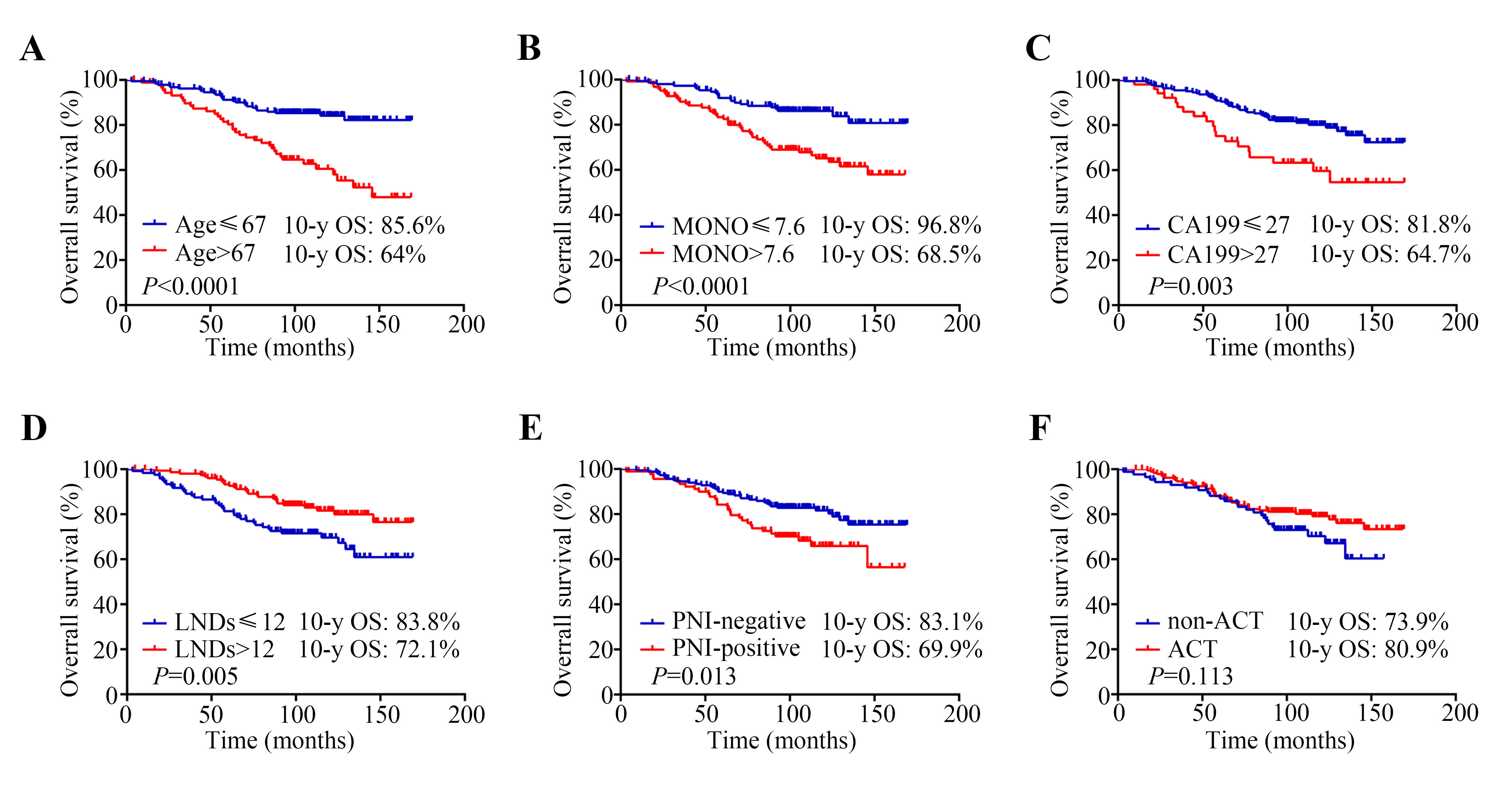

Supplement: Supplementary Figure 1 — Kaplan-Meier analysis of overall survival according to (A) age (≤ 67 vs. > 67 years, 10-y OS: 85.6% vs. 64%, P < 0.0001); (B) monocyte percentage (MONO%) (≤ 7.6% vs. > 7.6%, 10-year OS: 96.8% vs. 68.5%, P < 0.0001); (C) carbohydrate antigen 19-9 (CA199) (≤27 U/ml vs. > 27 U/ml, 10-year OS: 81.8% vs. 64.7%, P = 0.003); (D) lymph node dissection numbers (LNDs) (≤12 vs. > 12, 10-year OS: 72.1% vs. 83.8%, P = 0.005); (E) perineural invasion (PNI) (negative vs. positive, 10-year OS: 83.1% vs. 69.9%, P = 0.013); (F) adjuvant chemotherapy (ACT) (non-ACT vs. ACT, 10-year OS: 73.9% vs. 80.9%, P = 0.113). [file Image_1.TIF]

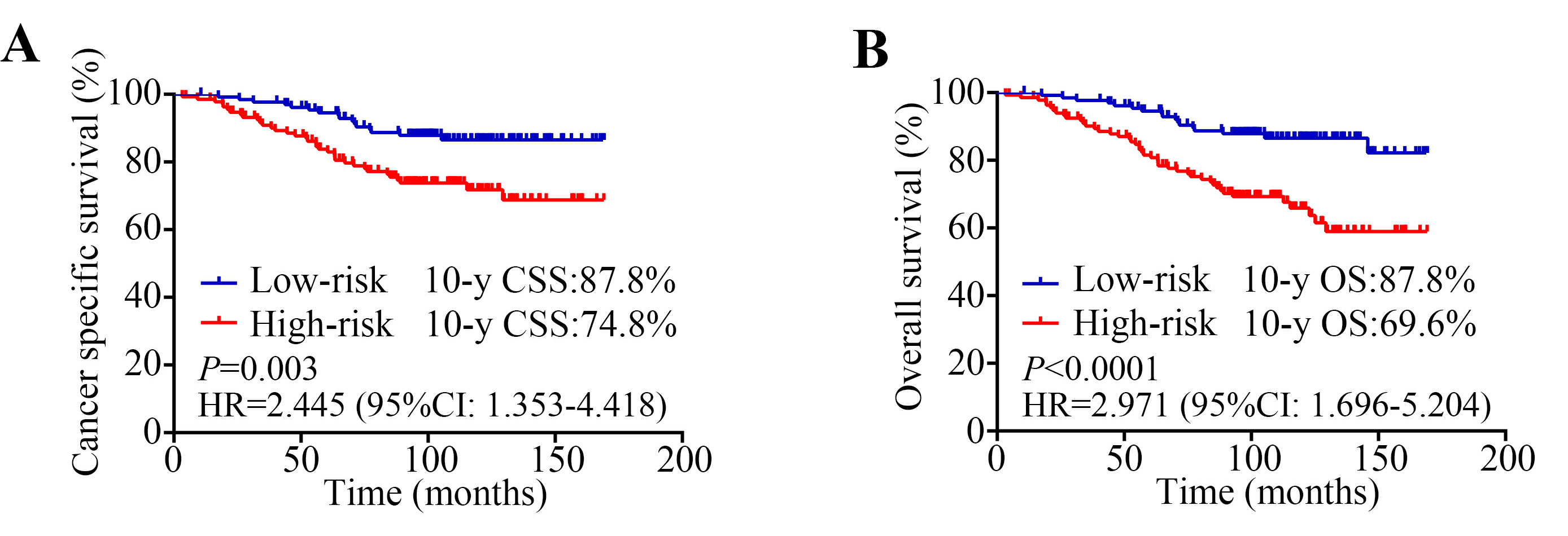

Supplement: Supplementary Figure 2 — Kaplan-Meier analysis estimates of the previous risk-stratification model (17) in our patients. (A) Cancer specific survival according to risk stratifications (low-risk vs. high-risk, 10-year CSS: 87.8% vs. 74.8%, HR = 2.445, 95%CI: 1.353–4.418, P = 0.003); (B) overall survival according to risk stratifications (low-risk vs. high-risk, 10-y OS: 87.8% vs. 69.6%, HR = 2.971, 95% CI: 1.696–5.204, P < 0.0001). [file Image_2.TIF]

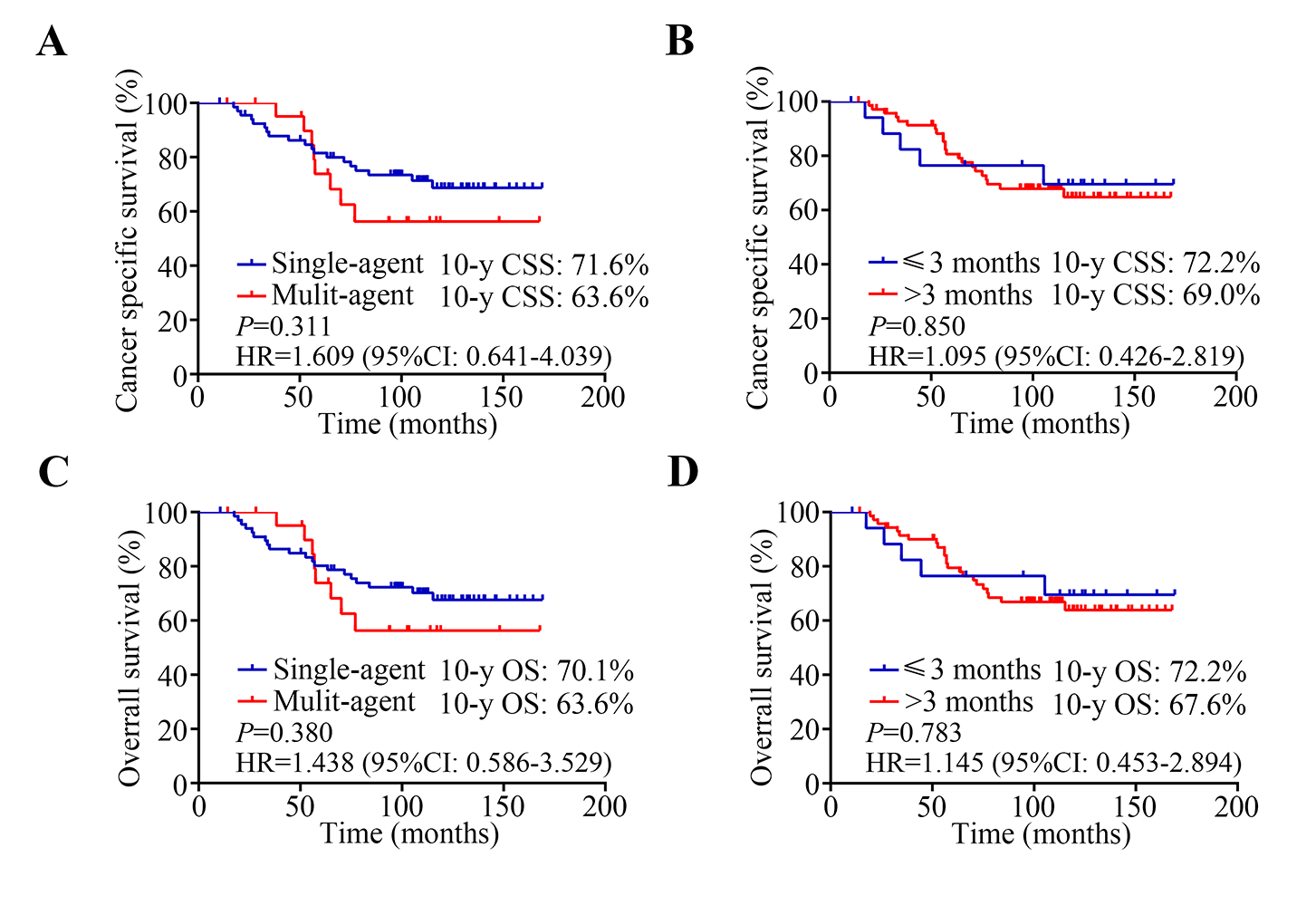

Supplement: Supplementary Figure 3 — Kaplan-Meier analysis estimates for high-risk patients. Cancer specific survival according to (A) adjuvant chemotherapy (ACT) for single-agent chemotherapy vs. multi-agent chemotherapy regimen, (10-year CSS: 71.6% vs. 63.6%, HR = 1.609, 95%CI: 0.641–4.039, P = 0.311); (B) ACT for 3 months or less (≤3 months) vs. more than 3 months (>3 months), (10-year CSS: 72.2% vs. 69.0%, HR = 1.095, 95%CI: 0.426–2.819, P = 0.850); Overall survival according to (C) ACT for single-agent chemotherapy vs. multi-agent chemotherapy regimen, 10-year OS: 70.1% vs. 63.6%, HR = 1.438, 95% CI: 0.586–3.529, P = 0.380); (D) ACT for ≤ 3 months vs. >3 months, (10-year OS: 72.2% vs. 67.6%, HR = 1.145, 95% CI: 0.453–2.894, P = 0.783). [file Image_3.TIF]
